# Development of VariLeg, an exoskeleton with variable stiffness actuation: first results and user evaluation from the CYBATHLON 2016

**DOI:** 10.1186/s12984-018-0360-4

**Published:** 2018-03-13

**Authors:** Stefan O. Schrade, Katrin Dätwyler, Marius Stücheli, Kathrin Studer, Daniel-Alexander Türk, Mirko Meboldt, Roger Gassert, Olivier Lambercy

**Affiliations:** 1 Rehabilitation Engineering Laboratory, ETH Zurich, Lengghalde 5, Zurich, 8092 Switzerland; 2 Product Development Group Zurich, ETH Zurich, Leonhardstrasse 21, Zurich, 8092 Switzerland

**Keywords:** Powered exoskeleton, Spinal cord injury, Overground walking, Powered gait orthosis, Wearable robotics, Variable stiffness actuation, Variable impedance actuation, Exoskeleton training

## Abstract

**Background:**

Powered exoskeletons are a promising approach to restore the ability to walk after spinal cord injury (SCI). However, current exoskeletons remain limited in their walking speed and ability to support tasks of daily living, such as stair climbing or overcoming ramps. Moreover, training progress for such advanced mobility tasks is rarely reported in literature. The work presented here aims to demonstrate the basic functionality of the VariLeg exoskeleton and its ability to enable people with motor complete SCI to perform mobility tasks of daily life.

**Methods:**

VariLeg is a novel powered lower limb exoskeleton that enables adjustments to the compliance in the leg, with the objective of improving the robustness of walking on uneven terrain. This is achieved by an actuation system with variable mechanical stiffness in the knee joint, which was validated through test bench experiments. The feasibility and usability of the exoskeleton was tested with two paraplegic users with motor complete thoracic lesions at Th4 and Th12. The users trained three times a week, in 60 min sessions over four months with the aim of participating in the CYBATHLON 2016 competition, which served as a field test for the usability of the exoskeleton. The progress on basic walking skills and on advanced mobility tasks such as incline walking and stair climbing is reported. Within this first study, the exoskeleton was used with a constant knee stiffness.

**Results:**

Test bench evaluation of the variable stiffness actuation system demonstrate that the stiffness could be rendered with an error lower than 30 Nm/rad. During training with the exoskeleton, both users acquired proficient skills in basic balancing, walking and slalom walking. In advanced mobility tasks, such as climbing ramps and stairs, only basic (needing support) to intermediate (able to perform task independently in 25% of the attempts) skill levels were achieved. After 4 months of training, one user competed at the CYBATHLON 2016 and was able to perform 3 (stand-sit-stand, slalom and tilted path) out of 6 obstacles of the track. No adverse events occurred during the training or the competition.

**Conclusion:**

Demonstration of the applicability to restore ambulation for people with motor complete SCI was achieved. The CYBATHLON highlighted the importance of training and gaining experience in piloting an exoskeleton, which were just as important as the technical realization of the robot.

## Background

Every year, over 250 000 people experience a spinal cord injury (SCI) worldwide [1]. In the United States of America, the costs induced by SCI are estimated to be about $2.3 million over the lifetime of a person if the injury occurs by the age of 25 years [2]. About 40% of SCIs lead to paraplegia [2], leaving many people in need of assistive devices to regain mobility in their daily lives. Assistive mobility devices can help decrease healthcare related costs by improving users’ independence and increasing their productivity. So far, wheelchairs are the gold standard to restore mobility for people with no or very little walking capability. However, wheelchair users remain constrained, especially in their ability to overcome obstacles such as inclines and stairs, or uneven ground. The SCI population is typically confronted with secondary complications such as higher rates of infections, high blood pressure, neuropathic pain, pressure sores [3–5], social stigmatization, increased rates of depression [6, 7], and a shorter life expectancy [2], some of them being linked to a lack of physical activity and mobility. Hence, restoring the capability to walk is among the top priorities for many SCI survivors and healthcare professionals [8].

Powered lower limb exoskeletons are a promising solution to achieve independent walking, which could improve the quality of life by mitigating negative health consequences of prolonged sitting, enabling eye-to-eye contact with adults and increasing community participation [9]. Powered lower limb exoskeletons are robotic structures that can be attached to the legs and torso in order to verticalize the user and move the legs according to pre-programmed patterns. Balancing is usually not fully supported, which is why crutches are needed. These exoskeletons are mainly used for two applications in the SCI population. First, as therapeutic tools in rehabilitation clinics, where they are expected to increase training duration and intensity, and therefore support rehabilitation mostly of incomplete SCI patients to regain the ability to ambulate [9, 10]. Several studies reported that the regular use of an exoskeleton could have a positive impact on chronic neuropathic pain, emotional and psychological constitution [11], bowel and bladder function [11–13], and spasticity [9, 12–15]. After training, users were also able to improve the speed and duration of continuous walking close to limited community ambulation capabilities [11, 16]. It has been reported that users were able to ambulate at a level of exertion that leads to health benefits and yet does not result in early fatigue [17]. Second, exoskeletons can be used as assistive devices to support people in performing activities of daily living at home and enabling walking as a daily exercise. Despite the availability of several exoskeletons on the market [18–21], current devices typically only support walking on even terrain or, at most, climbing stairs. This limits their ability to maneuver in a real-life environments and situations. Further, existing devices are also limited in walking speed, which is typically around 0.26 m/s [22], whereas 0.44 m/s would be considered necessary to achieve limited community ambulation capacity [23] and 1.06 m/s to safely cross a street [24]. Research prototypes of powered exoskeletons have been proposed to overcome mobility barriers such as stairs or inclines [25–27]. However, there is little information about the usability and performance of these devices, and on how they should be used to train users with SCI.

Over the past few years, we have developed a novel powered exoskeleton, the VariLeg. The unique feature of the VariLeg is a variable mechanical stiffness actuation (VSA) unit that drives the knee joint. It is inspired by the human capability to adapt the joint stiffness to different phases of the gait cycle [28] and to external perturbations. This is thought to be a key component for the low energetic cost of transport of human walking compared to state-of-the-art bipedal robots [29]. Additionally, adjustable compliance is also expected to increase efficiency and robustness against falling on uneven terrain [30, 31]. Adaptable compliance (mechanically or through control) has proven to be a valuable addition to increase safety and stability of human-robot interaction in gait rehabilitation robotics and assistive devices [32, 33] such as the Lokomat [34], LOPES [35] and the C-Leg [36]. We hypothesize that such adaptable compliance in a powered exoskeleton could provide advantages to cope with uneven terrain, or external perturbations and increase the achievable gait speed by allowing more dynamic walking.

This paper reports on the design and evaluation of the VariLeg exoskeleton, from test bench measurements of the functionality of the VSA to training with two users with thoracic motor complete SCI who received weekly training sessions over a period of four months, in view of participating in the CYBATHLON 2016 [37]. The Powered Exoskeleton Race discipline of the CYBATHLON 2016 involved different tasks corresponding to typical activities of daily life (e.g. overcoming uneven terrain, walking curves or climbing stairs) [37, 38]. These should be completed in a minimal amount of time, as part of a championship for people with disabilities using advanced assistive devices.

The applicability and performance of the VariLeg exoskeleton during preparation and participation at the CYBATHLON 2016 were evaluated. This was achieved by investigating (i) the ability of the device to assist SCI users to walk and complete different tasks of relevance in daily living, (ii) the usability of the system by detailing the progress and challenges faced by users with SCI and no prior experience with mobile exoskeletons over the course of the training, as well as their subjective feedback on the device, and (iii) discuss and compare the overall performance of the participant with the VariLeg exoskeleton at the CYBATHLON 2016, which was considered as an objective field test for the system (i.e. operating in a non-laboratory environment and under time constraints). The performance at the competition, as well as the experience gathered during the training phase, were used as indicators of the applicability of the VariLeg exoskeleton as an assistive device supporting users in mobility tasks of daily life. Furthermore, the reported learnings may help other groups wishing to contribute to this challenging and fast growing field.

## Methods

### Concept of the VariLeg exoskeleton

**Fig. 1:**
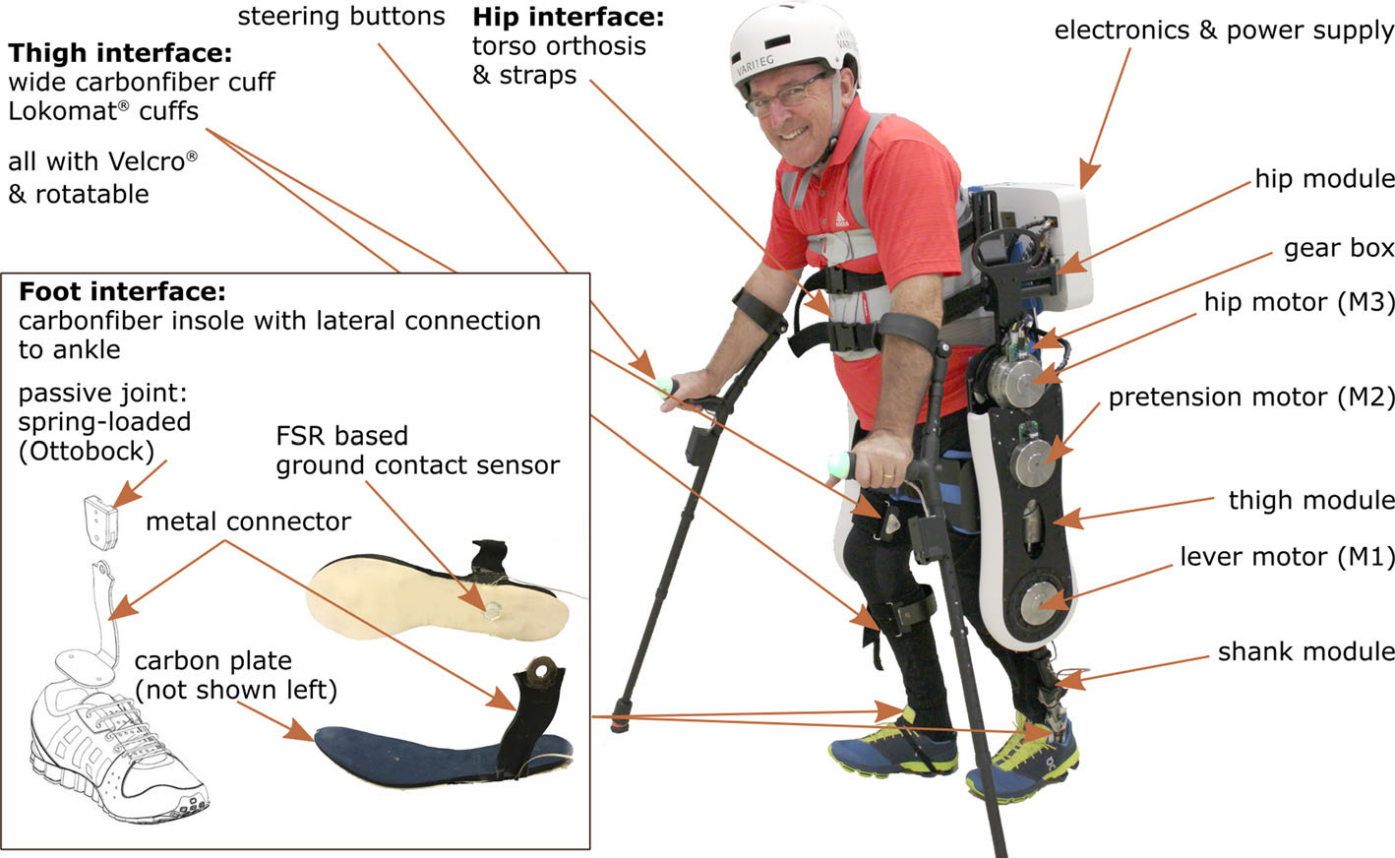
VariLeg exoskeleton with user (motor complete thoracic SCI). A variable stiffness actuator (VSA) in the knee joint can mimic the stiffness modulation observed in individuals with unimpaired gait (M2/M1). The hip joint is actuated conventionally with an electric motor and a reduction gear box (M3). Cuffs on the leg and a torso orthosis fix the exoskeleton to the user. The user balances using crutches that also serve to pilot the device through push buttons (e.g. triggering steps). Left inset: Details of the foot interface including a spring-loaded passive ankle and ground contact sensing

The VariLeg is a powered lower limb exoskeleton that restores walking ability even for users with a complete loss of motor function e.g. due to SCI (Fig. 1). As it was designed primarily as an assistive device for users with a thoracic motor complete SCI, the exoskeleton was intended to perform mobility tasks of daily life such as overcoming stairs or master uneven ground, while supporting the full body weight of the user.

The robotic structure has three degrees of freedom in the sagittal plane in each leg, two active for hip and knee flexion/extension, and one passive for ankle flexion/extension. The exoskeleton is attached to the user via cuffs at the leg, and a torso orthosis. Crutches are used for balance and as a user input interface.

The motors (EC90 flat, maxon motor AG, Switzerland) can deliver a maximum continuous torque of 0.56 Nm, while having a maximum speed of 3120 rpm. They are driving the joints through a transmission (SHD-25-160-2SH-SP, Harmonic Drive, Germany) with a reduction ratio of 1:160. This results in a continuous torque of 89.6 Nm at the transmission output with a maximum speed of 19.5 rpm.

A commercial spring-loaded passive ankle joint (Unilateral ankle joint 17LA3, Ottobock, Germany) was chosen over an actuated ankle joint to reduce the complexity of the exoskeleton and minimize the weight at the end-points of the legs.

As walking is possible without active push-off at the ankle, the passive joint only has to provide toe lifting during swing phase and compliance on uneven surfaces.

The power supply board and the battery are stored in an electronic box attached to the hip frame of the exoskeleton. The battery (37 V/5000 mAh LiPo-battery, Swaytronic, Switzerland for the motors and 7.4 V/4000 mAh LiPo-battery, Swaytronic, Switzerland for the onboard computers) was dimensioned to support 1–2 hours of operation depending on the performed task. The VariLeg contains a main computer for high-level control (i.e. trajectory calculation) and three slave computers for low-level control of the motors (i.e. joint position control). The main computer (Intel Edison Development Platform, Intel Corporation, United States of America) and one of the three slave computers (STM32F4Discovery with customized pinout boards) are located in the electronic box. The other two slave computers are located in the two legs to reduce cabling complexity and to keep analog signal lines short. Covers (SLA parts made from Accura Xtreme, Müri Prototech, Switzerland) are placed outside the structure of the exoskeleton to cover sharp components and prevent any possible harm during transfer into and use of the exoskeleton.

A wide upper thigh cuff, custom-made from carbon fiber-reinforced plastic (CFRP), was used to prevent unwanted rotation of the user’s thigh relative to the exoskeleton’s thigh. The lower thigh and the shank cuffs are commercial cuffs from the gait rehabilitation robot Lokomat (Hocoma AG, Switzerland). The torso is attached via a commercial orthosis (Dorso Direxa Posture, Ottobock, Germany). At the level of the feet, customized CFRP shoe inserts are mounted to the ankle orthosis. Ground contact is detected using force sensitive resistors (FlexyForce A201, Tekscan, United States of America) on the shoe inserts, placed on the location corresponding to the heel. The crutches are modified Flexyfoot (Flexyfoot Ltd., United Kingdom) crutches equipped with a custom-made handle incorporating push buttons, which serve as a user input interface. The hip width and the thigh and shank lengths are adaptable to fit users with height between 1.75 m and 1.90 m, and weight up to 85 kg.

### Variable stiffness actuator

**Fig. 2:**
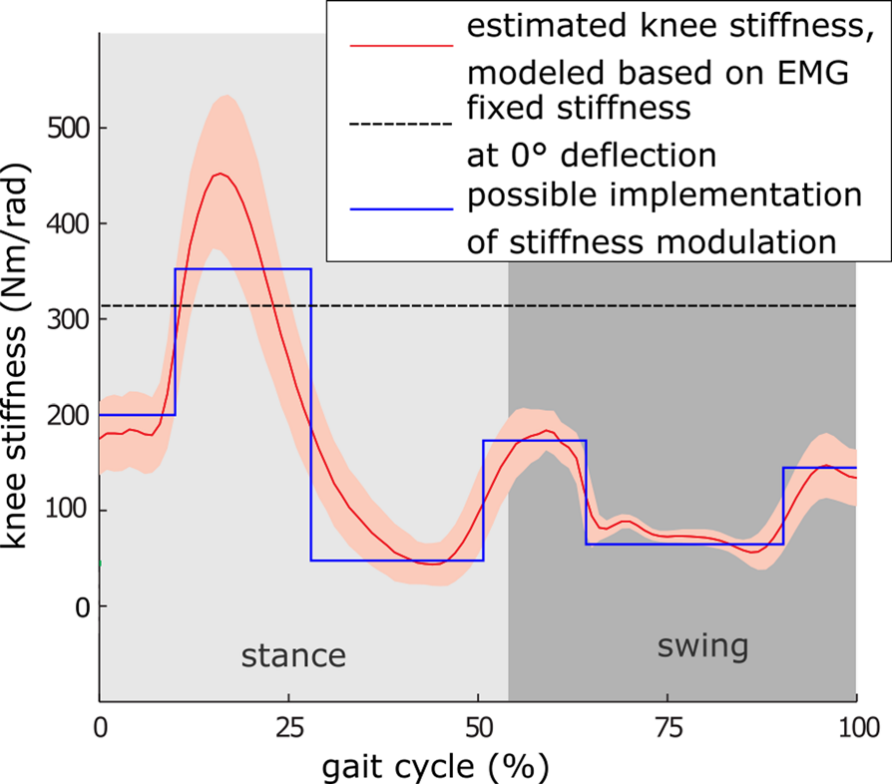
Stiffness modulation in the knee joint during gait. The expected human knee joint stiffness modulation during gait was estimated through an EMG-based model, which was verified in static (isometric) condition (adapted from [28]). A possible implementation of stiffness modulation could be to simplify this behavior into several regions with constant stiffness. The controller switches through these levels according to the gait phase. At the CYBATHLON 2016, we used a simpler strategy commanding a fixed stiffness setpoint. Nevertheless, the illustrated stiffness levels could be achieved in test bench experiments. Note that the gait cycle starts and ends with a heel strike of the same leg in this representation

It was desired that the actuator’s stiffness range would cover the expected stiffness modulation range of the human knee joint as closely as possible. The human stiffness modulation was estimated from an EMG-based model, which was verified in static conditions [28]. The VSA unit in the knee aims to imitate the human knee stiffness modulation during gait (Fig. 2), specifically, the high stiffness during stance, i.e. during early stance at heel-strike and during push-off at toe-off. This behavior observed in human walking presumably ensures effective load transmission properties when force is exchanged with the ground to decelerate (at heel-strike) or accelerate (at toe-off) the leg and the body’s center of mass. In swing phase, the leg is moving freely advancing as a pendulum. Besides the energetic benefits mimicking this strategy may have for robotic ambulation, it may also render collisions in swing phase less dangerous for the user and the robotic hardware, as the impacts are softened by a compliant behavior.

**Fig. 3:**
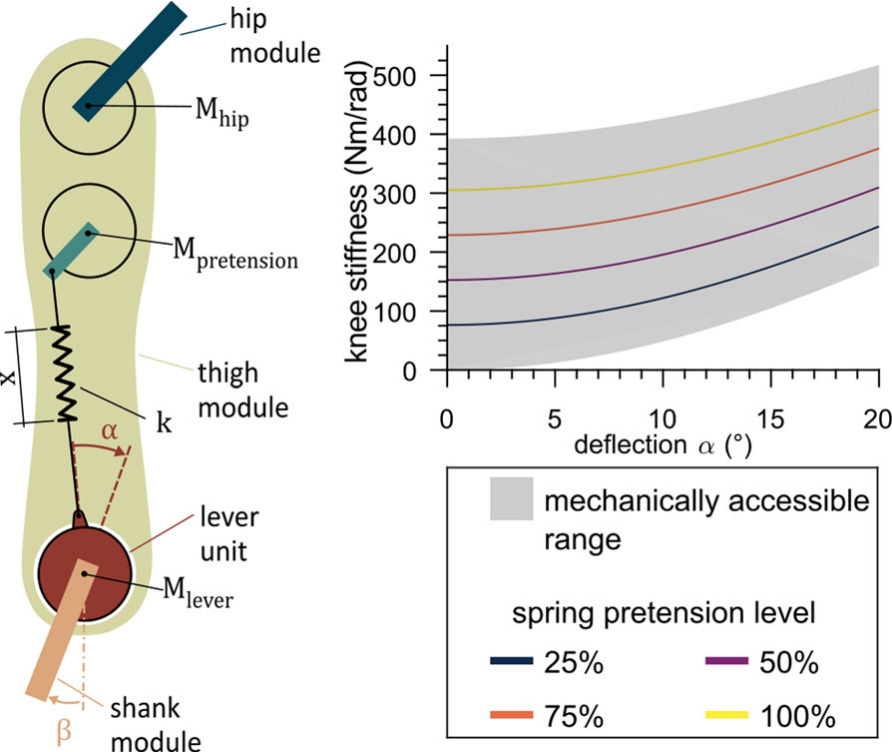
Schematic of the Variable Stiffness Actuation (VSA) unit and its expected stiffness range. The VSA (inspired from the MACCEPA and MARIONET systems) is illustrated on the left. The lever motor (*M*_*lever*_) situated in the lever unit controls the lever position relative to the shank. The lever unit is connected to the thigh through the spring *k*, which can be pretensioned (by the pretension motor *M*_*pretension*_). Varying pretension, which changes spring length *x*, results in a change of the stiffness. The stiffness also varies with the deflection *α*, describing the deflection of the lever unit from its equilibrium position. Stiffness in function of *x* and *α* is shown on the right. The mechanically available stiffness modulation range is indicated as a grey area. Holding a pretension continuously is limited by the motor’s continuous current limit indicated with the 100% line (yellow). The relative angle between thigh and shank (knee angle) *β* therefore depends on the lever’s equilibrium position, the load applied to the joint and its stiffness

The VSA in the knee joint is inspired by the MACCEPA [39] and the MARIONET [40] systems (Fig. 3), and was adapted to meet the specific size and output power requirements of the exoskeleton. It consists of two motors: one sets the equilibrium position of the shank relative to the lever unit. The other motor pretensions the spring (stiffness *k* = 109 N/mm) that connects the lever unit to the thigh. The more pretension *x*, the higher the stiffness (Fig. 3). As the VSA allows deflections (*α*) of the lever unit from its equilibrium position, the angle between the lever unit and the shank is not equal to the knee angle. Hence, a potentiometer at the knee additionally measures the angle between thigh and shank (*β*). This deflection is limited to 20° in both directions by the mechanical structure. Theoretically, a stiffness between 0 Nm/rad and 392 Nm/rad can be achieved at the equilibrium position (0° deflection). At maximum deflection, the stiffness can be varied between 177 Nm/rad and 518 Nm/rad. The maximum stiffness at equilibrium position is slightly lower than the maximally expected human knee stiffness. However, this compromise was chosen to keep the weight and torque requirements of the motors and its transmissions in reasonable ranges.

Due to the time limit given by the fixed date of the CYBATHLON 2016, the VSA was used with a fixed stiffness mode during the training and the competition (Fig. 2, dashed line). This was decided as we expected that learning to use an exoskeleton is easier as the device would act in a more predictable way than with a fixed stiffness compared to a device varying its stiffness. Additionally, development iterations to implement and test a suitable VSA control strategy would have required more time than the 4 months of training available until the start of the competition.

The VSA was evaluated for its ability to modulate stiffness on a test bench setup, which consisted of one single exoskeleton leg fixed to a metal test frame at the proximal end of the thigh and at the distal end of the shank. The continuous current rating of the motor limits the continuous pretension range to 0–0.028 m. In this range, four series of measurements were conducted with spring pretension levels of 25%, 50%, 75% and 100% of the maximum continuously achievable pretension level. With each pretension level, the lever motor was controlled to slowly move back and forth 5 times from -20° to 20° deflection with a constant velocity of 0.14 rad/s, while the lever motor current was measured. The motor current was filtered with a first order low-pass filter with cut-off frequency of 5 Hz during acquisition (ESCON Module 50/5, maxon motor AG, Switzerland). The current was converted into a torque estimate with the given torque constant of 0.109 Nm/A and the gear ratio of 160:1. This estimate was filtered offline with a second order low-pass Butterworth filter with a cut-off frequency of 10 Hz. The deflection angle *α* (see Fig. 3) was calculated by subtracting the knee angle *β*, defined as the angle of the shank relative to the thigh (Potentiometer 533B1103JC, Vishay, United States of America), from the lever unit angle, defined as the lever position relative to the shank (Potentiometer 3590S-6-103L, Bourns, United States of America). Torque as a function of deflection angle was fitted with a third order polynomial. The derivative of this fit was used as the stiffness estimate. The theoretically expected torque and stiffness for a given deflection angle were calculated using the equations derived by Van Ham et al. [39], adapted to the dimensions of our mechanism. Experimental data were then compared to the theoretical curves by calculating the root mean squared error (RMSE) between the fit of the experimental data and the corresponding theoretical values.

### Control

The control architecture of the VariLeg exoskeleton relies on low- and high-level controllers [41]. A position controller is implemented at the level of each motor (low-level control) to adjust joint angles according to predefined trajectories. The PID gains were manually tuned to minimize rise time without displaying overshoot. The high-level control computes stiffness setpoints (for example as proposed in Fig. 2) and trajectories resulting in reference joint positions (*φ*_*rh*_ and *φ*_*rl*_) as well as desired pretension motor position (*φ*_*rp*_). All control loops run at 100 Hz.

Three modes with different joint position trajectories were implemented: (i) “walking”, which can perform forward and backward steps, (ii) “inclines” for walking up and down slopes and (iii) “stairs” for climbing up and down stairs. Additionally, the exoskeleton can perform sit-to-stand and stand-to-sit transitions.

**Fig. 4:**
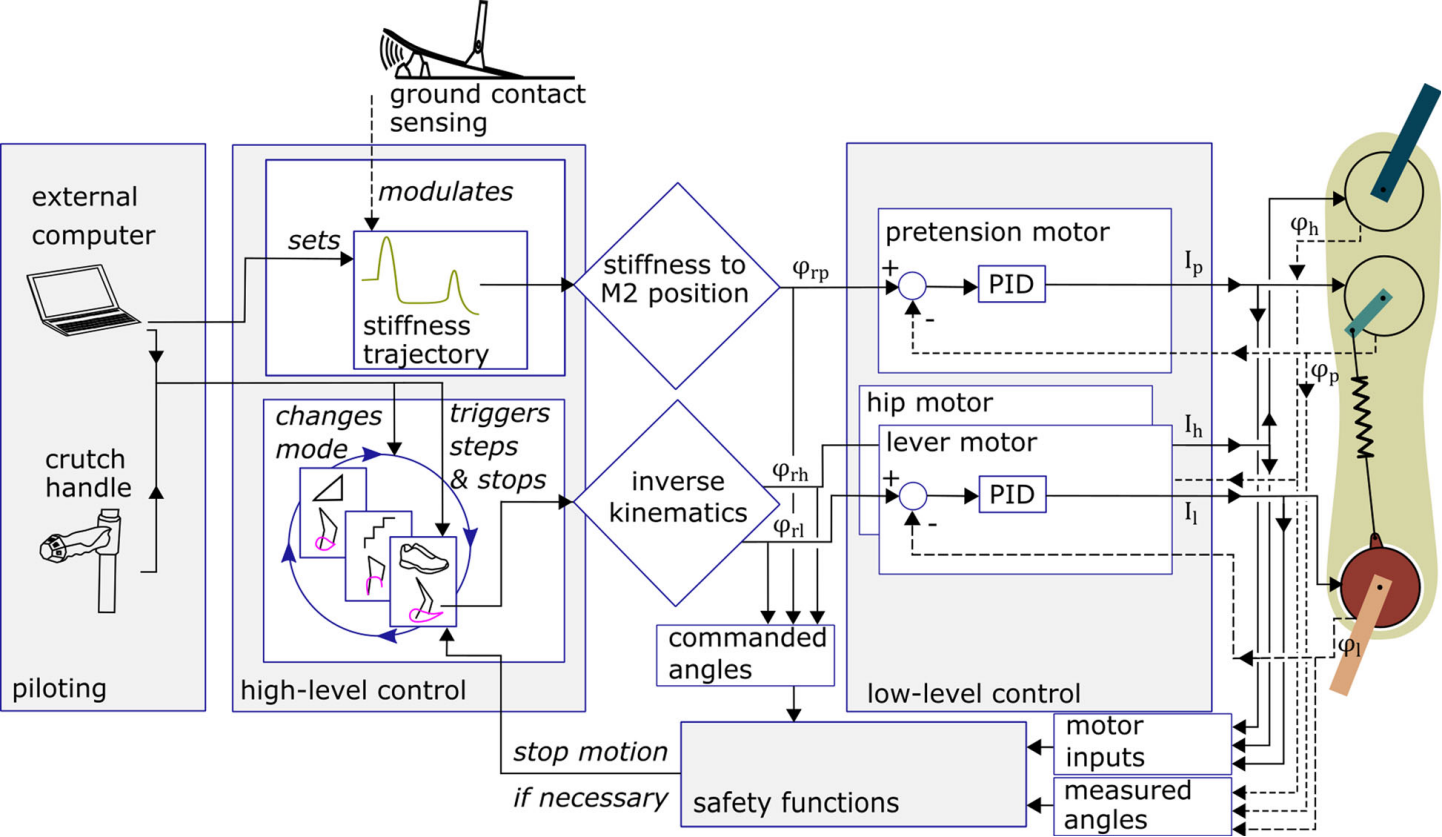
Overview of the control structure of the exoskeleton. The control architecture is divided into three parts: high-level control, low-level control and safety functions. The high-level control is replaying trajectories for the exoskeleton joint positions and the stiffness setpoint. The individual tasks have differring trajectories grouped in modes. The modes can be selected by the user pressing buttons on the crutches or by an operator with an external computer. The trajectories are executed by a low-level position control loop for each joint. The exoskeleton state is supervised by safety functions that stop the exoskeleton if, e.g., the redundant sensing disagrees or the motors receive a position request that is outside of the allowed range of motion. *φ*_*rl*_, *φ*_*rp*_, *φ*_*rh*_ designate the reference joint angles, defined by the trajectories (stiffness for *φ*_*rp*_ and walking, inclines or stairs respectively for *φ*_*rl*_ and *φ*_*rh*_). *φ*_*l*_, *φ*_*p*_ and *φ*_*h*_ are the angles measured with the position sensors that are fed back to the low-level controller and evaluated in the safety functions of the exoskeleton. *I*_*l*_, *I*_*p*_, *I*_*h*_ designate the current sent to the motor. *l* refers to the lever, *h* to the hip and *p* to the pretension motors

The exoskeleton can be piloted via push buttons on the left and right crutch handles. The user triggers the steps individually with a button on the handle of the right crutch. After each step, the user can decide to return to parallel stance or trigger another step. When standing with both feet parallel, the user can switch between modes (Fig. 4) or sit down. They also allow to adjust the step length and pause the movement at any time. Alternatively, the exoskeleton can be piloted over an external computer that is connected to the exoskeleton wirelessly, e.g., for early training or testing.

**Fig. 5:**
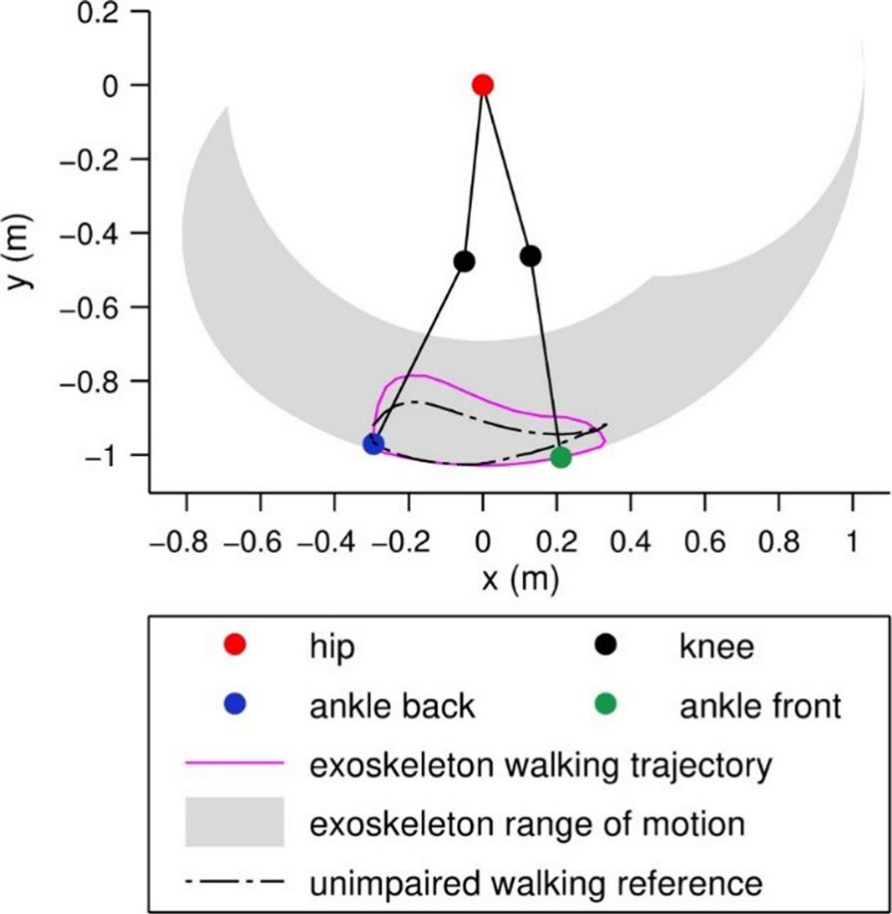
Walking trajectory of the exoskeleton compared to unimpaired gait. The nominal exoskeleton walking trajectory commands the equilibrium position of the knee more towards extension in early stance compared to unimpaired gait. This ensures buckling occurs due to the compliance of the VSA when loaded and is not pre-programmed into the trajectory. Ground clearance of the swing leg was increased to prevent collisions of the foot with the ground

**Fig. 6:**
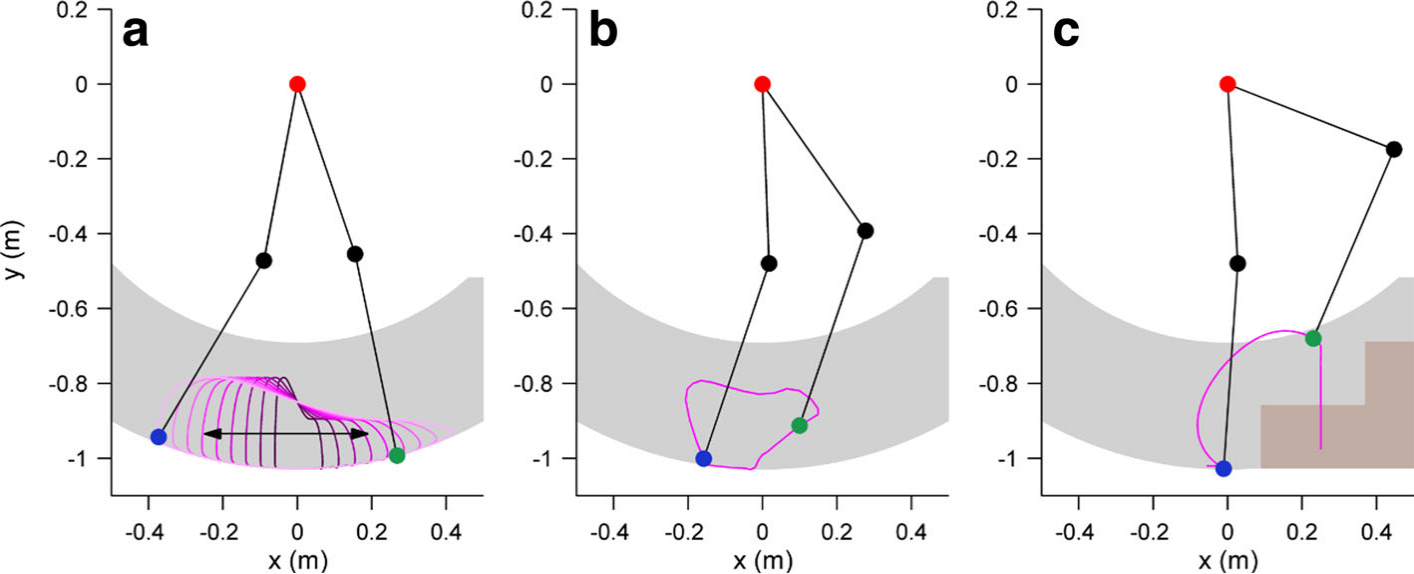
Walking scaling, incline and stair climbing trajectories of the VariLeg exoskeleton. The walking trajectories can be scaled in length (shown in **a**) and height to adjust the step. Different trajectories for walking, inclines or stairs were implemented and can be selected via the crutch or a computer wirelessly connected to the exoskeleton. The incline trajectory (**b**) was created by rotating the walking trajectory and prolonging the knee extension during late stance. The stairs mode (**c**) climbs steps one foot at a time and measures the height of the first executed step, which is performed at maximal step height

The nominal exoskeleton walking trajectory was based on reference data from unimpaired human walking [42] with some modifications. Stance phase knee flexion, which is thought to enable smoother load transfer from one leg to the other in double stance, was not pre-programmed in the knee angle trajectory. Rather it was left to occur as a result of the inherent compliance (Fig. 5). The ground clearance of the swing leg was additionally increased to prevent collision of the foot with the ground, which could lead to a premature end of the step, and even destabilize the user. The steps can be scaled in length and height (Fig. 6a). The latter provides adaptable ground clearance, which is useful for novice users: clearance was initially set high and was decreased with experience to allow more efficient walking. Length scaling influences walking speed, together with the replay speed of the trajectory.

Another possibility is to alter the replay speed of the trajectory.

The incline trajectory was defined by rotating the reference trajectory for walking and prolonging knee extension during early stance. The user can adjust the trajectory rotation with the buttons on the crutches for slopes between -20° and 20° (Fig. 6b). This allows to overcome inclines encountered in daily living and the ramp obstacle of the CYBATHLON track.

The stair-climbing mode is implemented in two phases. First, the initial step performs a forward movement of the right foot with maximal ground clearance and moves it down until ground contact is detected. The stair height is then computed from the configuration of the exoskeleton segments. In the second phase, the computed height is used to automatically adapt the reference trajectory, and bring the left foot next to the right foot. This reference trajectory with adapted height is then used for all the subsequent steps triggered by the user (Fig. 6c). An analogous procedure is used to walk down the stairs.

### Safety

Safety of powered exoskeletons is critical, as paraplegic users typically cannot perceive and provide feedback on pain or discomfort. As a first step towards this goal, an extensive Failure Modes and Effects Analysis (FMEA) was performed to systematically identify and assess all the possible risks of injury. The FMEA was used to quantify the risks based on three predefined categories: Severity, Occurrence and Detection. Different approaches were used to make the list of risks as complete as possible. First, various perspectives were considered to identify risks: user, supporting staff, and engineer. During this process, all the interactions these groups of people could have with the system, together with their inherent risks were identified. Next, various system failures that could lead to a health risk were identified. Last, injuries that could occur were listed and it was checked where in the exoskeleton and how they could arise. Where necessary, countermeasures were defined to minimize the identified risks.

Requirements that had to be fulfilled by crucial system components were specified. These requirements for software, hardware and electronics were verified with a series of tests derived from the FMEA. Verification started on the component level, continuing to the sub-assembly level and finally ended on the system level.

This resulted in three system layers for safety: software, electronics and mechanical. The software layer includes checking redundant sensor inputs and congruence of motor input commands with changes in sensor feedback, avoidance of unallowed joint angles to prevent joint overstretching, monitoring battery supply voltage, and limitation of angular velocity and torque. All software safety features are implemented in the low-level control, allowing for easy changes of the high-level control (e.g., implementation of new or adapted trajectories) without compromising safety. In addition to redundant sensing, the electronic safety layer consists of an independent power supply to the computers and to the motors. The independent power supply allows immediate shutdown of the motors in case of emergency without cutting the power to the on-board computers. This enables continued data recording to investigate the cause of the problem. The power to the motors can be switched off by two independent emergency shutdown buttons at the back of the exoskeleton. When power is cut off, the exoskeleton collapses and the supporting staff has to guide the user and the robot softly to the ground. The mechanical safety layer consists of mechanical end stops at the actuated joints to prevent joint overstretching if all other safety layers fail. Handles placed on either side of the exoskeleton allow staff to hold on to the exoskeleton and manually support it in case of an emergency or when the user loses balance.

The staff leading and supporting the training sessions with the exoskeleton were considered as a last safety layer additional to the technical safety mechanisms. Thorough instructions were given to all supporting staff, who were accompanying users and intervened if necessary. Their instructions included information on where to touch the exoskeleton, how to support the user, and how to react in case of emergency. This included a standard operating procedure covering reactions to all potential incidents identified during the FMEA, guaranteeing efficient and adequate actions even under stress. The instructions were followed by a practical training on how to shut down the system in emergency situations by cutting the power to the motors and subsequently guiding the user softly to the ground. This was practiced several times with an unimpaired user in the exoskeleton before the supporting staff was cleared to support or supervise training sessions. After hardware or software changes, the exoskeleton was always tested with unimpaired users before allowing users with paraplegia to use the device.

### User Selection

Two persons with SCI were recruited to test the applicability and usability of the VariLeg exoskeleton. Their role was to test the system, provide feedback for fast design iterations, and finally, for one of them, to participate in the CYBATHLON 2016.

Inclusion criteria for users consisted of:

- Spinal cord injury at thoracic or lumbar level, leading to leg paraplegia classified as AIS^1^ A or B, with a complete loss of motor function
- Sufficient voluntary control of trunk, arms and neck to keep the trunk and head upright and to use crutches to balance
- More than one year post-injury
- More than 18 years of age and able to give informed consent

Exclusion criteria were:

- Any restriction in the range of motion of the ankle, knee or hip
- Dizziness during transfers, standing training and similar situations
- Any injury or disease that could interfere with the training (e.g. shoulder problems)
- Weakness in the upper body or poor general fitness level

Additional practical criteria including time availability and transport to training locations were considered. Detailed information about the two recruited users are found in Table 1.

**Table 1:** Information on users testing the VariLeg exoskeleton

Specifications	Unit	User 1	User 2
Age	Years	40	57
Height	m	1.83	1.77
Weight	kg	78	85
Sex		Male	Male
Years post injury	Years	7	3
Lesion height		Th 4	Th 12
AIS classification		B	A
Self-reported	Low/moderate spasms		None
clinical syndromes			
Previous experience	Lokomat (Hocoma		None
with exoskeletons	AG, Switzerland)		

### Training and participation in the CYBATHLON

The exoskeleton prototype was designed to perform tasks of daily living such as overcoming inclines and stairs. However, before performing these advanced mobility tasks, standing and basic walking skills needed to be acquired. The targeted training schedule for testing the VariLeg was set to three sessions a week over four months with each session lasting 60 minutes. This time does not include preparing the exoskeleton, transferring into the system and donning or doffing. The training sessions were evenly distributed over the week. The training period was defined by the availability of the prototype and the set date of the CYBATHLON.

The training period consisted of three parts: (i) adjusting the exoskeleton, (ii) acquiring basic balancing, standing and walking skills, and (iii) training advanced mobility tasks.

The exoskeleton fitting and donning procedures are similar to the ones described by Asselin et al. [38]. Before the first training session, a physical therapist measured the joints’ range of motion and the length of the thigh (lateral condyle of knee to greater trochanter) and shank (lateral malleolus to lateral condyle of knee), and the pelvis width (left greater trochanter to right greater trochanter). These anatomical measures were used to adjust the segment lengths and the attachment system of the exoskeleton. The adjustment and fit of the attachment system were checked before every training session, as misalignment between the body and the exoskeleton could lead to unwanted loading of the musculoskeletal system. The first two sessions were dedicated to the evaluation of the user attachment system, ensuring it was safe for the following training sessions. In the first training session, the rotational joints axes of both, the user and the exoskeleton, were aligned after the user transferred from the wheelchair into the sitting exoskeleton. Users remained seated in the exoskeleton for 20 min. No standing or walking was performed in this session to avoid the risk of decubitus. We decided to advance slowly in the beginning as people with SCI, in some cases, are unable to notice uncomfortable pressure points and the injury prolongs healing time of wounds [43]. After transferring back to their wheelchairs, their skin was checked for pressure marks. In the second session, users stood up with the help of the exoskeleton and stood for ten minutes. The exoskeleton was suspended from a custom-made body weight support system (BWSS) consisting of a metal frame on wheels to help the user balance and prevent falls. After sitting down, they were checked for pressure marks again. In general, checks of the skin for pressure marks were performed after each training. Users were also instructed to check their skin at home with the help of their spouse or medical staff.

After the first steps in the BWSS, a walker was used before finally using crutches. The walking aids were changed according to user’s skills and preferences throughout the course of the training. Balancing in the exoskeleton was trained as soon as users switched to crutches to minimize the need for staff support and the reliance on the walking aids. Balance training consisted of standing upright and shifting weight in different directions. Users were encouraged to attempt maximal weight shift before supporting staff had to intervene to prevent falling. This allowed users to get a feeling for the dimensions and the weight of the exoskeleton. Once crutches were used, sit-to-stand and stand-to-sit transitions were also practiced until users were able to perform them independently.

More advanced mobility tasks such as climbing stairs, ramps and maneuvering uneven ground were addressed as users felt comfortable with walking. These advanced mobility tasks were trained in order of increasing difficulty, starting with varying the step length. Users were encouraged to identify the longest step possible. Making curves on a slalom course was trained before walking up inclines, maneuvering over uneven ground and climbing stairs. The incline training started with slopes of about 10°, which were increased to 15° until a maximum slope of 20° was climbed. Maneuvering uneven ground was trained by walking on pathways with inclines to the side (frontal plane of the user).

During every session, two supporting staff, one on each side, physically supported the user during the learning of new tasks, preventing falls in case the user lost balance. Each task was initially performed with physical support and instructions of the staff. As users improved, the physical support was decreased from holding and leading the exoskeleton in the beginning to just being in reach to catch or support the user when necessary. A third person was in charge of monitoring the state of the exoskeleton, and piloting and stopping the device remotely in emergency situations. This person could also trigger steps allowing the user to focus on the movement of the exoskeleton. This was frequently used when new tasks were introduced.

For evaluation purposes, the skills that were acquired during the training period were classified into four categories: basic, intermediate, advanced and proficient. The evaluation was performed by the supporting staff after training a task. Basic skills are achieved when users can perform the task with the physical support of staff but not when unsupported. Intermediate skills are achieved when the task can be completed independently with a success rate of at least 25%, with support required at least temporarily in the other attempts. Advanced skills require the user to complete the task in 75% of the attempts without help. Proficient skills stand for independent completion.

Finally, the CYBATHLON 2016 championship served as a field test to evaluate the performance of the VariLeg exoskeleton with a trained user. In particular, it allowed testing the exoskeleton in a non-laboratory environment, with the additional stress caused by the competition and spectators. For this purpose, the dimensions of the obstacles used during training were similar to the ones selected for the CYBATHLON track [37].

## Results

### Exoskeleton prototype

Following two years of development and testing, and iterations over two prototypes, a functioning powered exoskeleton was realized. Technical details on the exoskeleton can be found in Table 2. The batteries were dimensioned to last for 1.5 to 2 hours. This was expected to be sufficient to complete training sessions while keeping the weight added by the battery minimal. Tasks with high energy and torque demand such as stair climbing or repeated stand up and sit down can decrease the battery life to 1 hour. Over 80 potential failures were analyzed, e. g., overstretching of the joints, which is prevented by the mechanical stoppers in the joint, or injury of supporting staff by getting clamped by the exoskeleton, which is prevented by thoroughly and systematically instructing the supporting staff where it is safe to touch the exoskeleton. Consequently, more than 100 tests were performed to minimize the risks associated with the use of the exoskeleton. As an example, the mechanical stops were tested to withstand twice the nominal torque of the motors.

**Table 2:** Technical specifications, typical training preparation time and walking speed of the VariLeg prototype

Specifications	Unit	
Weight	kg	35
Max. cont. joint torque	Nm	89.6
Max. joint velocity	rpm	19.5
Max. knee joint stiffness	Nm/rad	380
Battery life	h	1.5–2
Typical preparation time	Min	30
Typical don/doff time	Min	10
Typical walking speed	m/s	0.2
Typical stride frequency	Hz	3
Maximum step length	m	0.5
Hip width	m	0.345 – 0.400
Thigh length	m	0.435 – 0.495
Shank length	m	0.380 – 0.440

**Fig. 7:**
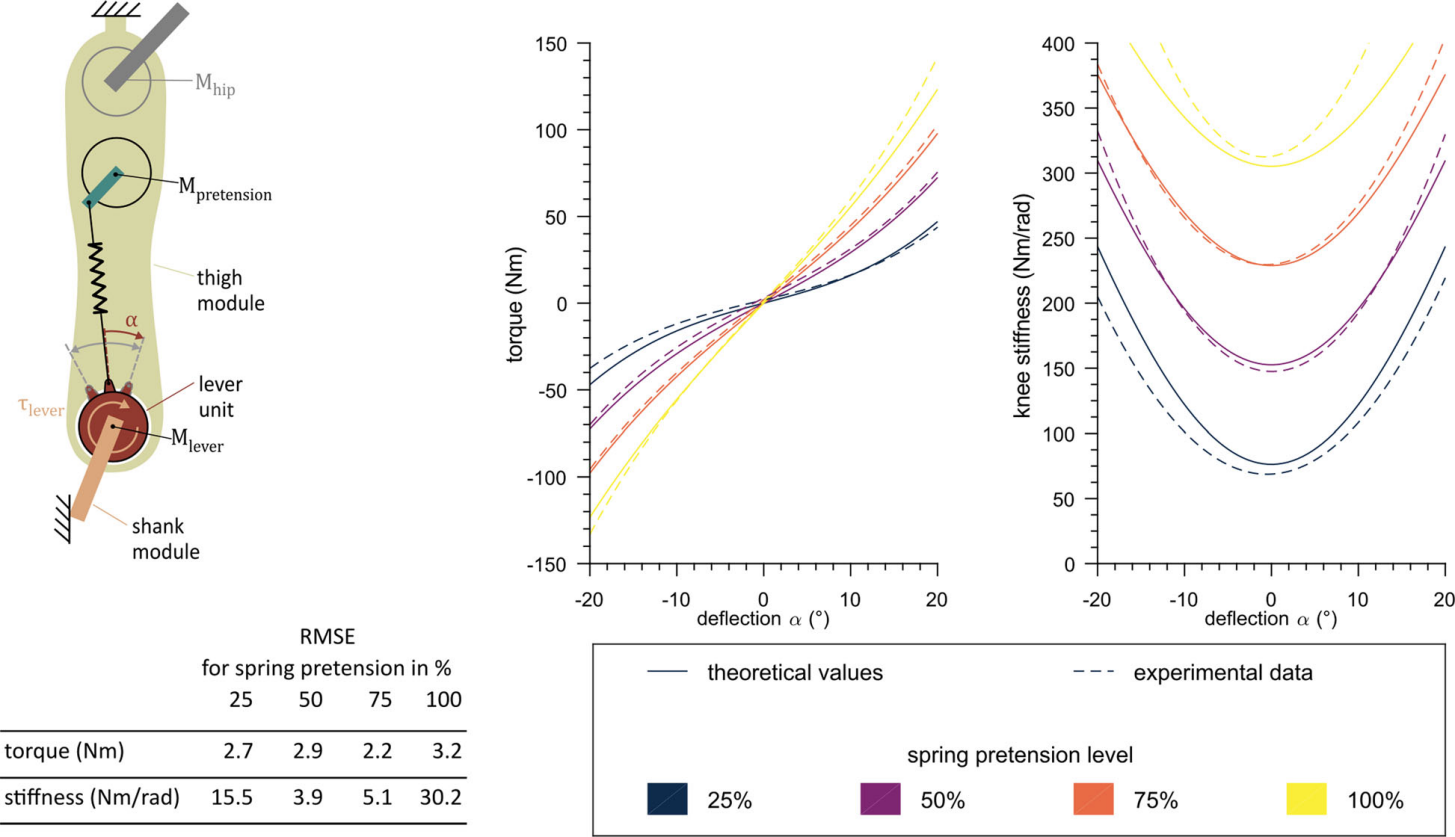
Results from MACCEPA characterization. Experimental results were compared to theoretical values. Stiffness is higher for higher deflections at high pretensions. Experimental torque fits match theoretical data within 2 to 3 Nm RMSE, whereas stiffness curves display larger errors of up to 30 Nm/rad deviation at the highest pretension

The VSA unit in the knee joint was evaluated on a test bench setup. The torque and stiffness over deflection for spring pretension levels of 25%, 50%, 75% and 100% of the nominal range are displayed in Fig. 7. The RMSE between the theoretically expected curves and the experimental data were between 2 and 3 Nm over a torque range of approximately -100 Nm to 100 Nm. Stiffness curves derived by numerical differentiation of the torque fit displayed larger RMSE especially for the lowest and highest pretension settings.

### Training

Users 1 and 2 completed 43 and 52 training sessions of 60 min duration, respectively. About 80 additional hours were needed to prepare the 95 training sessions.

No adverse events occurred during the training or the competition. Specifically, no falls occurred, but the supporting staff prevented three falls as users lost balance. No major skin irritations occurred. A small pressure mark (diameter of 1 mm) was observed on the foot of user 2, and disappeared after a week. It was not clear if the pressure mark was caused by the training or some other activity.

As users had no prior experience with powered mobile exoskeletons, they reported that the first few training sessions were needed to trust the exoskeleton and the supporting staff. The BWSS was only used for the first 3 training sessions. User 1 changed from the BWSS directly to crutches, while user 2 changed from the BWSS to a walker and, 6 training sessions later, to crutches.

Walking distance and speed increased with training. Approximately 5 meters of walking could be achieved before a rest was needed by users 1 and 2 after 2 and 5 training sessions, respectively. The progress was also slow in the beginning as the duration of a training session was limited by adjustment and setup time, as well as technical difficulties with the system. After 8 training sessions user 1 was able to complete a distance of 120 to 180 meters before sitting down again to rest. User 2 walked this distance without resting after about 15 training sessions. The maximum step length users could comfortably execute was about 50 cm. Walking speed after approximately 10 sessions was around 0.2 m/s, measured in a 10 m walking test which was completed in 47 s and 49 s, for users 1 and 2, respectively.

**Fig. 8:**
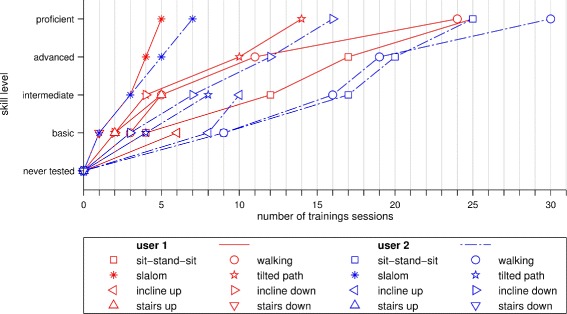
Amount of training necessary to achieve skill levels for different tasks. Both users required a considerable number of training sessions to gain proficient walking skills. The sit-to-stand motion was mastered after more than 20 sessions. Only basic skills were acquired on stairs and ramps

Figure 8 presents the training progress for the different tasks and obstacles for both users. User 1 achieved a proficient skill level on sit-to-stand, the slalom walking and tilted path tasks. He achieved basic skills in climbing up stairs, whereas climbing down the stairs was only performed once with the help of the supporting staff. User 2 achieved proficient level in slalom walking and sit-to-stand tasks. Only user 2 achieved an advanced skill level on the inclines.

### Performance at the CYBATHLON

**Fig. 9:**
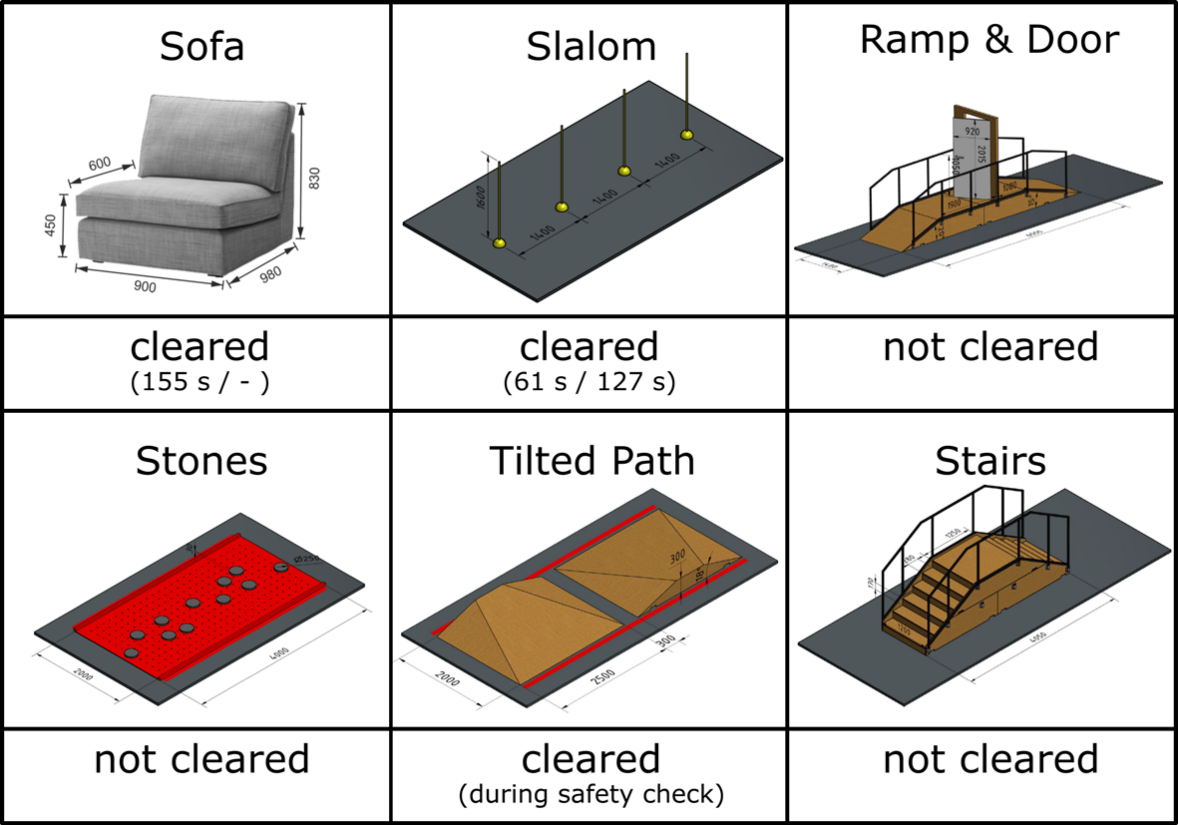
Performance of the VariLeg exoskeleton at the CYBATHLON 2016. The CYBATHLON 2016 obstacles presented in order of appearance during the championship (from left to right, top to bottom). Official time for clearance are indicated for the first and second run, if available. The sofa and the slalom obstacles could be cleared during the competition. The tilted path was only cleared during the safety check (i.e., the official test run prior to the competition)

User 1 competed at the CYBATHLON 2016^2^ and was able to sit down on and stand up from a sofa, walk a slalom in addition to, during the safety check (i.e., the official test run prior to the competition), cross the tilted path (Fig. 9). This corresponds to 3 out of 6 obstacles of the competition and resulted in 5^*th*^ place behind one commercial product and three research prototypes [25–27]. Flat stones, the ramp and the stairs were not attempted as there was not enough time to practice these obstacles before the competition, hence the user did not reach a sufficient skill level to complete them independently.

## Discussion

This paper presented the concept and design of the VariLeg exoskeleton, a unique lower limb powered exoskeleton with a variable stiffness actuator in the knee joint. This work aimed at establishing basic functionality of the prototype when used by people with a motor complete SCI. It reported on the progress two paraplegic users achieved on mobility tasks, walking distance and speed. The tests and training involved a learning process, in which physical exhaustion and caution of users towards the technology and helpers dominated in the beginning. In the final phase, the exoskeleton became the limitation as users increased their skill level.

### Exoskeleton prototype

The VariLeg exoskeleton proposed a novel concept for actively modulating knee stiffness online during gait. The capability of the VSA to vary stiffness by changing the pretension of the spring was evaluated on a test bench setup. A stiffness up to 450 Nm/rad, corresponding to the mean peak stiffness expected in human walking [28], can be achieved at deflection angles of 14°. However, this corresponds to a torque acting on the knee of 110 Nm, which is high compared to knee torques of around 45 Nm [44] during early stance of human gait (for a 1.8 m tall man with 85 kg body weight roughly corresponding to our users). This suggests that a stiffness of 450 Nm/rad was probably not applied yet despite the loads occurring in early stance. However, with varying stiffness, the pretension can temporarily be higher than the continuous torque would allow. The maximum achievable pretension would need to be further evaluated, as it depends on its desired duration and the stride frequency. Due to the time constraint imposed by the participation in the CYBATHLON 2016 championship, the VSA has not yet been used to modulate joint stiffness during walking. Instead, a fixed spring pretension was chosen for the training and the competition with a setpoint resulting in a stiffness of 305 Nm/rad at 0° deflection angle (corresponding to the 100% pretension curve in Fig. 3). Compared to the ALTACRO gait orthosis [45], which also uses a MACCEPA but is a stationary exoskeleton, our implementation offers more torque capacity and higher maximal stiffness. It is nevertheless not yet clear how these parameters influence performance in intended use, as the ALTACRO was not tested with paraplegic users. It is expected that more dynamic and more efficient walking could be achieved by further exploiting the VSA [30, 46]. This should also lead to increased stability on uneven ground, resulting in smaller forces necessary to balance with the crutches [31]. Without the possibility to vary compliance, the exoskeleton strictly defines the leg orientation independently of the ground property and the user has to adapt with his trunk to compensate for the uneven ground. An alternative to relying on the user for compensation would be a more intelligent controller that detects the properties of the environment and adapts its strategy accordingly. However, this would require increased sensing capabilities and computing power to process, analyze and react to different situations. Additionally, it would be more challenging to test and demonstrate the safety of an adaptive controller due to its complex behavior.

The implementation of a suitable controller remains to be investigated. It may be beneficial to adapt the stiffness variation strategy based on speed and body weight of the user, as has been observed in unimpaired walking [47]. Instead of a continuously varying stiffness profile, the modulation could be approximated by several regions of constant stiffness (i.e., setpoints, Fig. 2). Similar to what has been attempted in prostheses, it might be possible to use center of pressure information to modulate stiffness in synchronization with the gait cycle [48].

Aligning an exoskeleton to the user is a well-known challenge. Some groups suggested passive joints to prevent misalignment [49, 50], while others expect truly ergonomic devices custom-made for individual users in the future [51]. As in most currently available lower limb exoskeletons, the VariLeg used neither approach, but offered the adjustment of the user attachment system to segment dimensions. However, despite taking anatomical measures of shank length, thigh length and pelvis width in advance, several training sessions were needed to optimize the adjustment of the exoskeleton to each user. It was important to ensure that the joint axes of the exoskeleton coincided as closely as possible with the joint axes of the user to minimize shear forces, which could cause non-physiological loading of joints and bones, or skin abrasion. The risk of pressure marks was minimized by using padded attachment points, and by thorough visual inspection of the attachment before each training. Folds in trousers and socks fabric presented potential causes for pressure marks as well. With the current attachment system, users often displayed increased hip flexion during standing due to the non-adjustable plate on the hip frame supporting the pelvis. This structure should be improved to better support hip extension and possibly be adjusted to the individual body physique of users. We also observed that the user’s knee were more flexed during stance than the exoskeleton’s. We hypothesize that this is mainly due to the design of the cuffs on the thigh and shank, which have a more rigid part on the posterior side and allow some movement on the anterior side due to the elasticity of the straps. The current shoe inserts attached to the exoskeleton were not well suited for walking up inclines as the user’s foot often slipped out of the shoe. We suspect that the insoles were too stiff, which also prevented users from shifting their body weight anteriorly, e.g. when needed during standing up.

We expect that similar challenges are present when using other exoskeletons, although they are seldom reported especially in devices for the lower limbs [52–55], and quantifying them in a standardized way is not established yet [56]. Some studies have reported pain ratings in lower limb exoskeletons [13, 14], but it is important to note that they can only be evaluated for the body regions with unimpaired sensation or SCI users with residual sensory function.

### Training

The tests conducted with two users with SCI demonstrated basic functionality of the device for performing tasks of daily living. The robot was used frequently over an extended period of time, totaling 95 training sessions of 60 min duration until the CYBATHLON. The encouraging feedback collected from users and supporting staff during the training sessions informed the further improvement of the prototype (e.g. attachment system and improved trajectory control).

Sit-stand-sit transition and walking capability were first restored with the exoskeleton. Second, advanced mobility tasks such as overcoming stairs and inclines were attempted. Due to the limited time available for the training before CYBATHLON 2016, the functionality of the prototype could not yet be established for independent completion of all tasks with the prototype, but the achieved results are encouraging.

Training session frequency and duration was comparable to what others reported when training SCI users to pilot a powered exoskeleton [17] with a session duration of 60 min and a 3 times per week schedule. The training period of four months was rather long compared to other studies found in literature, where it varied between 1 and 24 weeks (see [17] for a review). However, most of these studies also used a commercial exoskeleton or a prototype in a very late development phase, while the VariLeg was still in development and undergoing improvements between the training sessions. It was helpful to train balance in the exoskeleton by having users shift their weight from foot to foot, and front to back while standing. This is also recommended by others [9, 38], as it increased awareness of the user on how to best balance with the additional weight of the exoskeleton while being upright in order to minimize the reliance on the crutches. The possibility to change the step length was helpful as a way to adjust gait as users gained experience and improved their performance. Stride frequencies of about 0.3 Hz helped users shift their weight from side to side, which was also reported to feel more natural than slower walking. Accordingly, it is not recommended to reduce step frequency but rather reduce step size if slower walking is desired. Tasks of daily living were targeted after basic training of balancing and walking, which was not always the case in other studies. While some studies performed relevant tasks beyond walking on an optional basis, like going to a cafe and standing upright while cooking [57], others added walking outdoors to the list of tasks [58]. Climbing stairs was investigated in studies with the ReWalk exoskeleton [57]. Overcoming inclines steeper than 8° or paths tilted in the frontal plane were so far not reported. The tasks and obstacles of the CYBATHLON required different amounts of training for the two users. Some tasks could be trained until a proficient skill level was reached, allowing the user to complete them without any help, whereas other tasks could only be trained to a basic skill level, enabling the user to successfully complete the task independently in about 50% of the cases. As a limitation, it has to be considered that the assessment of the skill level was subjective to some degree, as it was not based on clinical assessments and no fixed protocol was followed to assess the skill.

Preparation for training sessions took a similar time as the session itself. As more than 80 hours were used to prepare the 95 sessions with both users, the importance of considering the usability when designing an exoskeleton is evident. In comparison, typical preparation time with the Ekso^TM^, a commercialized exoskeleton, is 10 to 30 min with an average of 18.13 min [14]. Future development should consider shortening the preparation time, as this may be a key factor for the acceptance of assistive devices.

The training phase also allowed us to gather important information on key points to consider when training a novice user in an exoskeleton such as the VariLeg. An important factor for training success was the user’s confidence in the exoskeleton. This could be improved by presenting the robot, explaining how it works and showing it in action. Users gained confidence after seeing that they have control over the exoskeleton, being able to influence its behavior at all times. The supporting staff were crucial for trust and safety as they were able to catch the user and prevent falls if necessary. This staff has to be trained in handling the exoskeleton and how to react in case of system failure or fall. In addition, users should be trained on how to behave in case of such an incidence. Supporting staff should practice tasks in the exoskeleton for gaining experience to lead and instruct users when teaching new tasks.

### Participation in the CYBATHLON 2016

Competing at the CYBATHLON 2016 was a great experience for the whole team including our test users. Three out of six obstacles of the track could be completed by the user in the exoskeleton during the competition; we are confident that it would be possible to complete most of the obstacles with two additional months of intense training and some minor improvements on the hardware. The stairs were overcome repeatedly and successfully shortly after the competition. Inclines are expected to become easier to walk on with an improved design of the shoe inserts and improved motion trajectories. The exoskeleton prototype offers capabilities beyond overground walking that can extend the usefulness of such an assistive device for daily mobility. The long and extensive training also showed that there is still room for fine-tuning and that future exoskeleton users need to learn how the different features of the exoskeleton can be employed optimally in daily life situations.

In the following, we will briefly review each CYBATHLON obstacles in the order of which they appeared on the track, and discuss the performance of the VariLeg.

#### Sit-stand-sit

Standing up and sitting down was addressed well by the VariLeg exoskeleton. However, due to the very low height of the seat used at the CYBATHLON 2016 and the restrictions of the exoskeleton joint range of motion in the hip and knee, the crutches were needed to help stabilize and balance the user when standing up and sitting down. In combination with the slippery floor in the stadium, this rendered the obstacle much more strenuous than during the training. This illustrated that the use of crutches requires a ground that has good friction properties. If the crutches slip, proper piloting of current exoskeletons is not possible. If users fail to balance, this could lead to dangerous falls. The standing up motion of most exoskeletons is still slow compared to how people with no leg impairment stand up from a very low seat. Exoskeletons could be improved by performing a more dynamic and ballistic motion to optimally support the user and take the load off the arms.

#### Slalom walking

Walking curves for the slalom was possible even though the ab-/adduction movement was not enabled by the exoskeleton. The user could control the direction by pushing himself right and left with the crutches during swing in combination with leaning forward to establish ground contact earlier, rendering steps smaller when needed. This is where a compliant exoskeleton may have presented a benefit, as a stiff structure would supposedly make balancing with the crutches more difficult and strenuous, while establishing earlier ground contact. However, when walking curves, the exoskeleton did not support the user optimally. He had to direct the exoskeleton a lot with his arms in order to turn. This is not desirable as a long-term solution, as overloading of the arms could lead to secondary health issues in arms and shoulders. The compliance of the knee actuation may facilitate turning around the stance leg. This could be an alternative for an actuated hip ab-/adduction joint in the exoskeleton. Such a joint could provide rotational yaw torque to the structure when both feet are on the ground for turning and it could control the lateral foot placement during swing to support walking a curve. Both strategies could decrease the need for the user to push himself and the exoskeleton around his stance foot during swing to walk a curve. Ideally, balancing and walking without crutches would be possible. However, without an actuated ankle joint and actuated degrees of freedom in the frontal plane this can hardly be achieved. One of the reasons why only very few devices propose such designs is that it adds weight and complexity to the system [20, 59]. While the Mindwalker has not been able to allow people with SCI to walk without crutches yet, the REX can walk without crutches but only with a very static, hence slow, gait. Additionally, no work known to the authors has so far compared the necessary supporting forces in the crutches between actuated ab-/adduction and locked ab-/adduction.

#### Ramp

The ramp needed many training sessions due to the fact that the heel of the user was sliding out of the shoe when climbing the ramp. This lead to training interruptions until the shoe was correctly fixed to the user’s foot again. Inclines were considerably easier to descend than to ascend for users. Users were exhausted when walking up inclines, as they struggled to shift the center of mass over the feet alternately to ensure walking up the inclines and not just trotting in place. As it was difficult for users to walk up inclines, we also tried it using stair-mode, which turned out to be easier. Consequently, the strategy to generate optimal incline trajectories should be further investigated.

#### Flat stones

In the design process of the VariLeg exoskeleton, it was decided to focus on the implementation of a realistic assistive device rather than a system optimized for the CYBATHLON 2016 obstacles. In that sense, the maximal possible step length was set to 50 cm, which should be sufficient for most tasks of daily living, but is insufficient to complete the flat stone obstacle (as the longest distance between stones is 60 cm). It is important to note that shifting the user’s weight from one foot to the other becomes very hard without an active ankle if the steps are too long during slow walking. The variable step length that can be changed over a button on the crutch handles should nevertheless be a suitable and useful tool for daily mobility as it allows turning in narrow spaces by reducing the step length. Further, precise foot placement in the flat stones obstacle has to be controlled by the user since the exoskeleton has neither the necessary control strategy nor sensing capabilities.

#### Tilted path

The tilted path could be successfully completed during the training and the safety check, but unfortunately not at the competition (due to technical issues). This illustrates that robustness was a challenge for research prototypes competing at the CYBATHLON 2016. Walking on uneven ground like the tilted path proved to be strenuous for users. As they need the crutches to balance, it is uncomfortable if they are on uneven height. Exoskeletons ideally should support the user by adapting its gait pattern to the ground properties. However, this would require means to measure or estimate ground inclination. We hope to offer some adaptiveness over the VSA in the knee joint, which should allow the exoskeleton to passively adjust to the unevenness. This passive capability should be leveraged in future development with active adaptation of the trajectories to the uneven ground.

#### Stairs

The exoskeleton was able to overcome stairs during training sessions, but this feature was ready only shortly before the competition. As a consequence, the user had climbed the stairs only four times and descended it once, which was not sufficient to attempt overcoming this obstacle at the CYBATHLON 2016. Users reported that descending the stairs facing downwards is psychologically the most demanding task as the fear of falling was very present in this situation. With training users gained confidence in the exoskeleton and learned how best behave to leverage its abilities, decreasing the fear of falling.

#### Time limit and effect of lesion level

Due to the still limited walking speed of exoskeletons, a limitation also pointed out in [22], the time constraint alone made a strict prioritization of tasks necessary, as not all of them could have been performed in the 10 min time limit imposed by the CYBATHLON Powered Exoskeleton race. Additionally, the competition was demanding and exhausting for the user, as he needed to actively work together with the exoskeleton to fulfill the tasks. The user who joined the competition with the VariLeg has a lesion at Th4 and therefore no control over a major part of his trunk muscles, which is likely to make it more challenging for him to control the exoskeleton compared to a user with a lower lesion, as for example user 2 with a lesion at Th12. However, despite the difference in lesion height, no apparent difference in performance was visible between the two users. This was likely due to the high level of fitness and personal motivation of user 1, which underlines the necessity to keep wheelchair users motivated to stay in good shape and perform physical exercises as part of their daily routine.

### Remaining challenges

The experience of the user on how an exoskeleton is best used will always be a vital parameter in the overall performance of the symbiotic combination of human and machine. Thus, it is important that users are trained effectively and efficiently. As a consequence, early testing with the target population is crucial and strong bonds to clinical experts and test users are vital for engineers to conceive an optimal design. As it is impossible to use the exoskeleton without prior training, clear instruction need to be provided by trained personnel for use in the clinics or at home. They should assure that walking in the exoskeleton is learned in a physiologically correct manner to prevent negative health consequences caused by walking with a bad posture. As an example, instructing staff needs to teach how to use the walking aids, especially the crutches, for optimal stability and performance. Using crutches as early as possible should be encouraged by the training supervisor to assure fast progress.

Many of the obstacles that were overcome with the current prototypes were only possible thanks to users supplementing the missing capabilities of the exoskeletons with their arms and the muscles of their torso and shoulders. In the future, this should ideally be improved such that people with both impaired leg and arm function are also able to benefit from this exoskeleton technology. Appropriate control of the VSA in the VariLeg exoskeleton (based on e.g. matching knee stiffness measurements for active gait in unimpaired subjects [60]) could help increase the ability of the exoskeleton to maneuver uneven grounds, thereby partially relieving the user. This offers the potential to increase the usability of exoskeletons and variety of achievable tasks they can support, which could lead to better acceptance of the devices among the SCI population and healthcare professionals.

Falling is another problem that is generally not addressed by most existing prototypes or commercially available systems. They offer no measures to prevent falling or mitigate its effects. The current solution is to have accompanying people that either intervene to prevent a fall or, in the worst case, at least help the person getting up again or getting out of the exoskeleton. Future developments should take into account strategies regarding how a fall on obstacles and even ground could be mitigated either by appropriate reaction of the exoskeleton or additional safety measures as, e.g., airbags. If a fall occurs, exoskeletons should also provide a strategy to stand up again, which is not possible nor foreseen in designs at the moment.

There have been no longitudinal studies to investigate long-term effects of using an exoskeleton. Single case studies report improvements in neuropathic pain [9] and spasticity [9, 13, 14]. They coincide with the unstructured subjective feedback we received from our two test users. However, high user expectations are typically not met due to the limited capabilities of current exoskeletons [57]. While this issue may decrease as exoskeletons become more robust and offer advanced capabilities, current limitations should be openly discussed with users in order to understand what can realistically be expected from current exoskeletons.

Apart from the athletic competition, it is important to note that the CYBATHLON acted as a catalyst for the development of this project. Setting a well-defined goal motivated users to participate in the training sessions and to compete at the CYBATHLON 2016. Additionally, it promoted the collaboration of engineers, clinicians and users of the exoskeleton. The contact between those parties is still existing and accelerating the development of exoskeletons that can make the translation from a laboratory setting to the clinics and the daily life of people with SCI.

## Conclusion

The intensive training and testing with the help of two users with SCI demonstrated the basic functionality of the VariLeg exoskeleton. Besides walking on even ground, users learned to perform sit-stand-sit transitions, maneuver in a slalom course and overcome uneven ground tilted in the frontal plane. This allowed to overcome three out of six obstacles at the CYBATHLON 2016. During the training sessions, ramps and stairs were overcome with the additional help of the supporting staff, with indications that they could be overcome independently with further training. The CYBATHLON 2016 suggested that the use of powered exoskeleton technology for activities of daily living is still demanding, and a number of improvements are required including the capability to maneuver uneven ground with more ease, which we hope to achieve by implementing more advanced control strategies that take full advantage of the VSA implemented in the VariLeg exoskeleton.

## Footnotes

1 American Spinal Injury Association Impairment Scale (AIS) classification as defined by [61]

2 Detailed rules for the championship: https://www.ethz.ch/content/dam/ethz/special-interest/conference-websites-dam/cybathlon-dam/documents/2016-08-10_Cybathlon_RacesRules.pdf
